# Digits Lost or Gained? Evidence for Pedal Evolution in the Dwarf Salamander Complex (*Eurycea,* Plethodontidae)

**DOI:** 10.1371/journal.pone.0037544

**Published:** 2012-05-23

**Authors:** Trip Lamb, David A. Beamer

**Affiliations:** 1 Department of Biology, East Carolina University, Greenville, North Carolina, United States of America; 2 Department of Mathematics and Sciences, Nash Community College, Rocky Mount, North Carolina, United States of America; Brigham Young University, United States of America

## Abstract

Change in digit number, particularly digit loss, has occurred repeatedly over the evolutionary history of tetrapods. Although digit loss has been documented among distantly related species of salamanders, it is relatively uncommon in this amphibian order. For example, reduction from five to four toes appears to have evolved just three times in the morphologically and ecologically diverse family Plethodontidae. Here we report a molecular phylogenetic analysis for one of these four-toed lineages – the *Eurycea quadridigitata* complex (dwarf salamanders) – emphasizing relationships to other species in the genus. A multilocus phylogeny reveals that dwarf salamanders are paraphyletic with respect to a complex of five-toed, paedomorphic *Eurycea* from the Edwards Plateau in Texas. We use this phylogeny to examine evolution of digit number within the dwarf−Edwards Plateau clade, testing contrasting hypotheses of digit loss (parallelism among dwarf salamanders) versus digit gain (re-evolution in the Edwards Plateau complex). Bayes factors analysis provides statistical support for a five-toed common ancestor at the dwarf-Edwards node, favoring, slightly, the parallelism hypothesis for digit loss. More importantly, our phylogenetic results pinpoint a rare event in the pedal evolution of plethodontid salamanders.

## Introduction

The evolutionary loss of one or more entire digits (digit loss) is well documented in tetrapods and serves as a long-standing exemplar of convergence [1], [2]. The ubiquity of digit loss in certain taxa, e.g., 62 times in 53 lineages of squamate reptiles [3], has been the focus of phylogeny-based comparative methods designed to address mechanisms and correlates of loss. These surveys have revealed general patterns of digit loss in the transition from lizard-like (four pentadactyl limbs) to snake-like body form, where digit loss correlates strongly with limb-size reduction [4], [5]. Remarkably, some squamate comparative studies also provide evidence for digit re-evolution [5]–[8].

Evolutionary patterns in digit loss have also been reported for amphibians, particularly among salamanders, order Caudata [9]. Although digit loss–relative to the ancestral complement of four fingers and five toes–is widespread taxonomically (five out of 10 families), the total number of salamander taxa having experienced such loss is fairly limited (Table 1). Nonetheless, Alberch and Gale [9] noted that digit loss appears to be associated with miniaturization and paedomorphosis. They proposed that digit loss accompanying miniaturization could arise from global developmental truncation whereas loss associated with paedomorphosis may reflect slower rates of cell proliferation. Miniaturization in salamanders, especially for species with larger genomes (within concomitantly large cells), can promote developmental constraints and novelties [10]. For example, the miniature plethodontid salamander *Thorius* has undergone a reduction in the number of cranial elements [11], [12]. Thus, digit loss could feasibly result from a small limb bud’s limited number of large cells falling below some minimal developmental threshold required to produce a complete set of digits [13], [14].

**Table 1:** Taxonomic distribution of digit reduction in the order Caudata.

Genus	Family	Species #	Reduced digit #
*Batrachuperus*	Hynobiidae	5	P 4
*Paradactylodon*	Hynobiidae	3	P 4
*Salamandrella*	Hynobiidae	2	P 4
*Necturus*	Proteidae	5	P 4
*Proteus*	Proteidae	1	M 3; P 2
*Pseudobranchus*	Sirenidae	2	M 3; P 0
*Siren*	Sirenidae	2	P 0
*Amphiuma*	Amphiumidae	3	M 1,2,3; P 1,2,3
*Batrachoseps*	Plethodontidae	20	P 4
*Eurycea*	Plethodontidae	2	P 4
*Hemidactylium*	Plethodontidae	1	P 4

M  =  manus, P  =  pes.

Compiling morphometric data on 203 caudate species (representing all 10 recognized families), Wiens and Hoverman [15] used phylogeny-based comparative analyses to test Alberch and Gale’s [9] predictions on digit loss. Although they identified certain trends, relationships were largely taxon dependent. For example, digit loss was not associated with absolute body size but rather evolutionary changes in body size and, even then, due mainly to the influence of a single genus (*Amphiuma*). Wiens and Hoverman [15] did detect a significant association between digit loss and paedomorphosis, though only for large, elongate species in exclusively paedomorphic families (Amphiumidae, Proteidae, Sirenidae). They also detected relationships between toe loss and absolute (and relative) hind limb size but noted that genome size did not appear to factor significantly in either association. No relationship was detected between the digit most commonly lost (fifth toe) and miniaturization or paedomorphosis. Overall, evolutionary patterns of caudate digit loss are far more ambiguous than those for squamate reptiles. And unlike squamates, digit re-evolution has not been reported for salamanders, despite one hypothesis for limb development that posits the evolution of novel digits [16].

Here we present a phylogeny for the plethodontid genus *Eurycea* that reveals a change in digit number among closely related species. Our molecular phylogenetic survey centers on relationships in the *E. quadridigitata* complex [17], a four-toed species group known as the dwarf salamanders. Based on analyses of nuclear and mitochondrial genes, we reject the monophyly of dwarf salamanders and provide instead strong support for their paraphyly relative to a five-toed species complex from the Edwards Plateau in Texas. We use our phylogeny to examine the evolution of digit number in *Eurycea*, testing the contrasting hypotheses of independent digit loss (parallelism among dwarf salamander lineages) versus digit gain (re-evolution in the Edwards Plateau complex).

## Methods

### Taxon and Gene Locus Sampling

**Figure 1:**
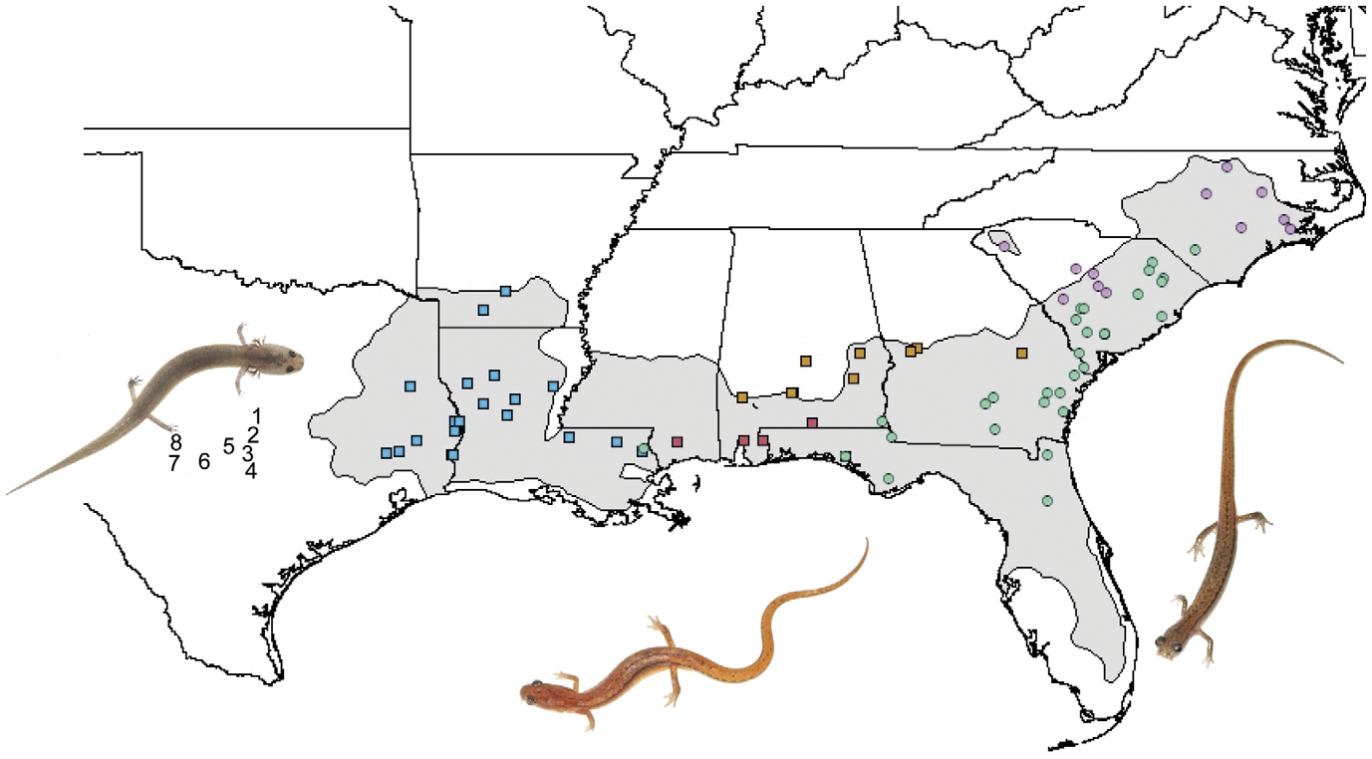
Distribution map of the dwarf salamander complex. Sampling localities are color-coded to depict phylogeographic lineage assignments (Fig. 2); numbers indicate localities for Edwards Plateau species (see Table S3). Illustrated, left to right, are *Eurycea tonkawae* (an Edwards Plateau species), *E. chamberlaini*, and *E. quadridigitata*.

The dwarf salamanders are one of the three plethodontid taxa characterized by loss of a single digit on the pes (Table 1). Distributed throughout the southeastern Coastal Plain, dwarf salamanders were considered to represent a single species, *Eurycea quadridigitata* [18], until a distinct color morph was elevated to species status (*E. chamberlaini*) [17]. Further, one of us (DAB) noted separate topological placements for eastern (South Carolina) versus western (Texas) *E. quadridigitata* in a phylogeny for the paedomorphic *Eurycea* that constitute the Edwards Plateau complex [19]. To examine lineage diversity among dwarf salamanders more fully, we generated DNA sequence data on 120 individuals, representing dense geographic sampling (88 localities, Table S1) across the range of the *E. quadridigitata* complex (Fig. 1). To explore phylogenetic relationships of dwarf lineages relative to the genus overall, we surveyed 15 additional species (representing the remaining four species complexes in *Eurycea*), including eight species in the Edwards Plateau complex (Fig. 1). Outgroup taxa included *Gyrinophilus porphyriticus*, *Pseudotriton ruber*, *Stereochilus marginatus,* and *Urspelerpes brucei* [20], which, together with *Eurycea*, represent all genera within the tribe Spelerpini [21]. Specimens were maintained and euthanized following standard procedures approved by East Carolina University’s Animal Care and Use Committee, outlined expressly for this survey (Animal Use Protocol # D247).

We sequenced portions of two mitochondrial genes–NADH dehydrogenase subunit 2 (*Nd2,* 1,020 bp) plus an adjacent transfer RNA (*tRNA^trp^*, ∼70 bp), and cytochrome *b* (*Cytb*, 1,012 bp)–for all specimens. We also sequenced three additional genes for a subset of dwarf salamanders (n = 23) representing phylogeographic lineages identified by our initial mtDNA dataset. These loci, chosen for slightly to substantially slower evolutionary rates relative to *Cytb,* included another mitochondrial gene, 16S ribosomal RNA (*16 s,* 529 bp), and two nuclear genes, pro-opiomelanocortin (*Pomc*, 536 bp) and recombination activating gene 1 (*Rag1*, 1131 bp). Amplification primer sets and cycling conditions are listed in Table S2. Sequences were generated on an Applied Biosystems 3130 capillary machine and aligned in CLUSTAL X 1.81 [22]. Protein-coding sequences were translated to ensure appropriate reading frames. Regions of the *16 s* alignment for which nucleotide position homologies varied across gap parameter settings were excluded, yielding a slightly smaller final dataset (512 bp). Genbank accession numbers are listed in Table S3.

### Phylogenetic Analysis

We analyzed two concatenated datasets (1 =  mitochondrial genes *Cytb*+*Nd2*+*tRNA^trp^*, and 2 =  all-genes) using Bayesian inference (BI) and likelihood (ML) methods. We identified nucleotide substitution models for each gene for BI, partitioning protein-coding genes by codon position and assessing gene/codon partitions by the Bayesian Information Criterion [23]. We implemented BI analysis in MrBayes 3.1.2 [24], [25], involving two concurrent runs of four simultaneous Markov Chain Monte Carlo (MCMC) chains for ten million generations, with a sample frequency of 1,000 generations. Topologies in the first 25% of the posterior distribution were discarded as burn-in, and the remaining trees were summarized as a majority consensus. Convergence of model parameters and topology were assessed by the program Are We There Yet (AWTY) [26].

ML analyses were executed in RAxMLHPC v7.2.8 [27], employing the rapid hill-climbing algorithm [28]. Parameters for the analyses incorporated the GTRGAMMA model of evolution, and 100 random addition sequence replicates were conducted. Branch support was computed via 100 non-parametric bootstrap replicates [29].

The degree to which individual gene sequences support (or are discordant with) clades identified by concatenated data reflects gene-tree heterogeneity. Concatenation approaches focused at the level of a species complex can generate misleading results due to incomplete lineage sorting, introgression, or deep coalescences [30]. In some of these cases, species tree inference methods (e.g., Bayesian concordance, coalescent models) can outperform data concatenation analyses [31]. Thus, we also estimated a species tree using a two-step Bayesian concordance analysis (BCA) [32] and a multispecies coalescent model, implemented in BEST ver. 2.3 [33].

For BCA, we generated posterior probability distributions for the gene tree of each locus separately using MrBayes 3.1.2 (4 MCMC chains; 5 million generations). Upon discarding the first 4 million generations from each locus run, we used BUCKy (Bayesian Untangling of Concordance Knots) v 1.2 b [34] to construct a primary concordance tree from the posterior distributions obtained for these loci. BUCKy also generates concordance factors, which represent the proportion of genes supporting a given clade. We conducted two BCA runs; in the first, mitochondrial genes were analyzed separately, for a total of five loci. In light of linkage, however, we combined the mitochondrial gene sequences in the second BCA, for a total of three loci (a single mitochondrial linkage unit; 2 nuclear genes).

We used BEST v 2.3 [33] to estimate a species tree that accounts for deep coalescence. Each phylogeographic lineage of the dwarf salamander complex was treated as a separate species for the BEST analysis, which ran for 120 million generations, with a sample frequency of 1000 generations. We used a uniform prior (0, 3) for gene mutation estimate and modeled the effective population size with an inverse gamma distribution (α = 3, β = 0.1). Convergence of model parameters and topology were assessed using AWTY [26].

### Ancestral State Reconstruction

We examined character state history of digit number (4 vs. 5 toes) using MCMC methods [35] implemented in the program BayesTraits V1.0 (www. evolution.rdg.ac.uk). To test the contrasting hypotheses of digit loss among the dwarf salamanders (parallelism) versus digit gain in the Edwards Plateau complex (re-evolution), we used the all-genes dataset to reconstruct the ancestral state for the node subtending the dwarf-Edwards clade. Reconstruction involved the reversible-jump model, with an exponential prior seeded from a uniform on the interval 0 to 30. We set the *ratedev* parameter to 8 (which, in conjunction with the previously identified prior, produced acceptance rates in the desirable (15–40%) range) and ran the analysis for 100 million iterations.

For a second assessment (again, using the all-genes dataset), we employed the fossilize command in BayesTraits, implementing two constraint analyses–the first fixing the dwarf-Edwards node at five toes (i.e., the parallelism hypothesis), the second at four toes (re-evolution hypothesis). We compared harmonic means for the two hypotheses using the Bayes factors statistic, where 2(lnL *H*_1_ − lnL *H*_2_) is the Bayes factor (BF), with a BF >2 interpreted as positive support and BF >5, strong support [36].

## Results

### Phylogenetic Analysis of Concatenated Sequences

**Figure 2:**
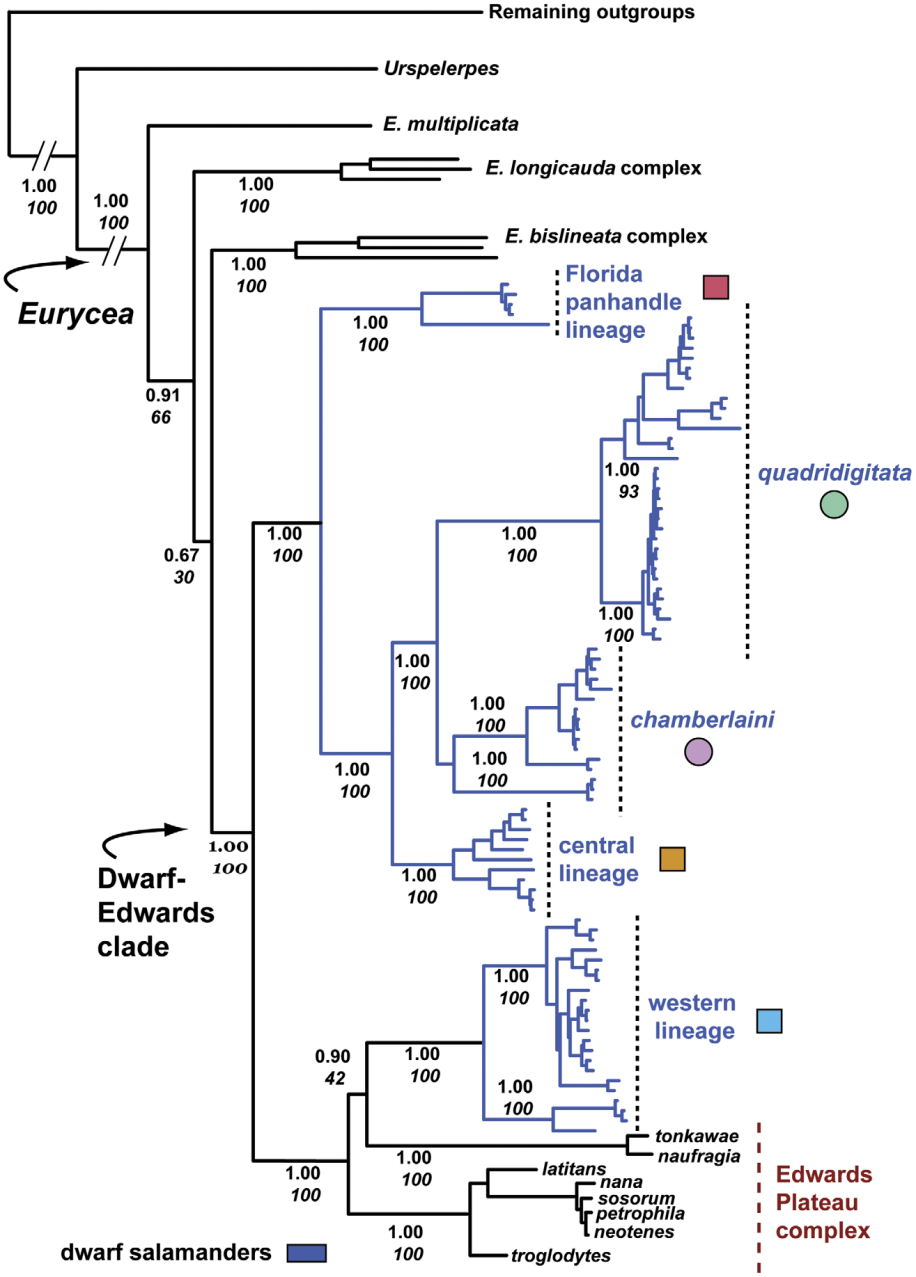
BI phylogram for the mitochondrial genes *Cytb*, *Nd2*, and *tRNA^trp^*. Numbers adjacent to nodes are Bayesian PP and ML bootstrap (italicized) values. Nodal support is not labeled for fine-scale branching within dwarf phylogeographic lineages (or other species complexes). Color-coded symbols accompanying dwarf lineages correspond to localities in Fig. 1. The outgroup species *Gyrinophilus porphyriticus*, *Pseudotriton ruber,* and *Stereochilus marginatus* are not shown.

We observed 87 haplotypes among 120 dwarf salamanders based on the mitochondrial genes (*Cytb*, *Nd2, tRNA^trp^*) dataset, from which we identified five phylogeographic lineages in BI and ML analyses. We refer the two eastern-most lineages to the currently recognized species *Eurycea quadridigitata* and *E. chamberlaini* (based on topotypic specimens) and designate the remaining three as the Florida panhandle, central, and western lineages (Fig. 2). Although the dwarf salamanders trace to a common node, they are paraphyletic by virtue of an additional group comprising eight species of paedomorphic *Eurycea* (each with five toes) from Texas (Fig. 2). As noted, the Texas paedomorphs represent a well-defined complex of 13 spring- and cave-dwelling species endemic to the Edwards Plateau and vicinity [19], [37], [38]. The dwarf salamander-Edwards Plateau clade (henceforth, dwarf−Edwards clade) receives strong support (Bayesian posterior probability [PP] = 1.0), as do its two subclades: 1) *quadridigitata* + *chamberlaini* + central + Florida panhandle lineages (PP = 1.0), and 2) western lineage + Edwards Plateau complex (PP = 1.0). Within the latter subclade, a sister group relationship occurs between the western lineage and two paedomorphs (*Eurycea naufragia* + *E. tonkawae*), which, in turn, forms the sister group to the remaining Edwards Plateau species (Fig. 2). The ML phylogram is identical topologically to the Bayesian consensus tree, with comparable levels of support (Fig. 2).

**Figure 3:**
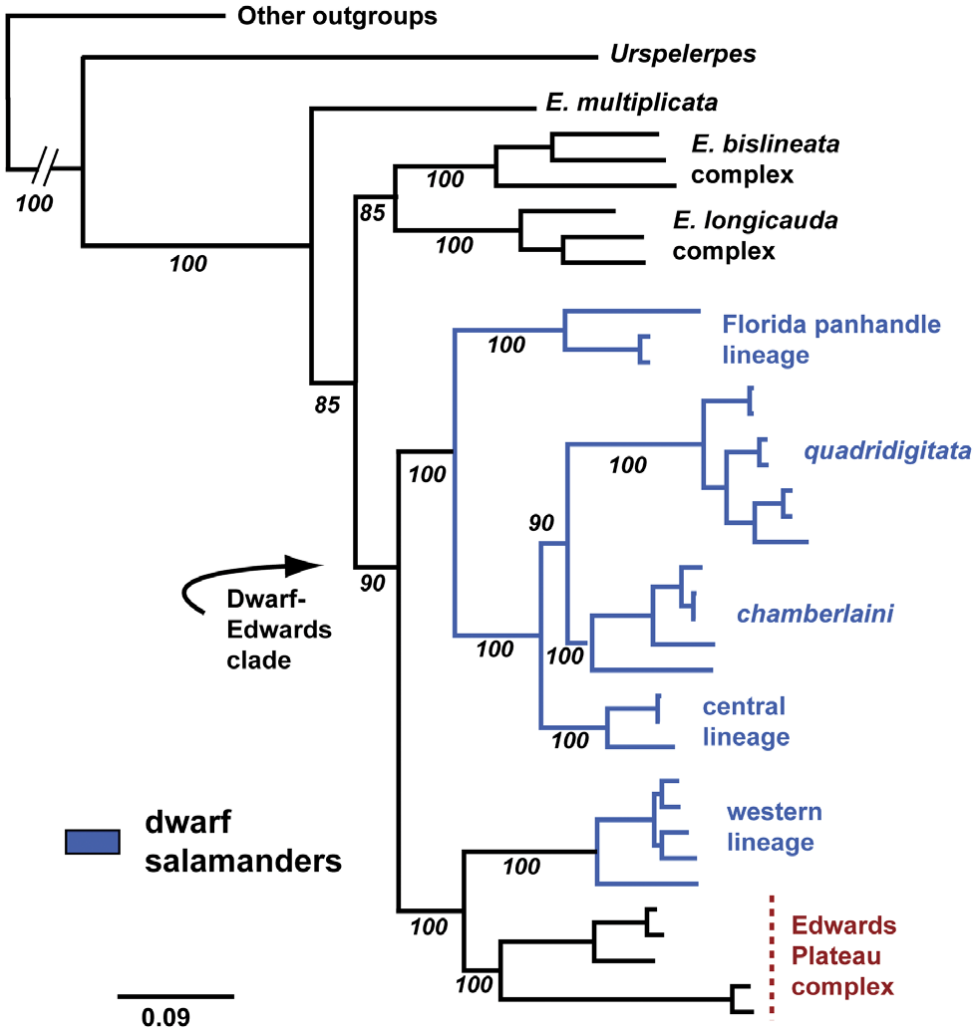
All-genes BI phylogram. All nodes have Bayesian PP values  = 1.00; ML bootstrap values are listed in italics. The outgroup species *Gyrinophilus porphyriticus, Pseudotriton ruber* and *Stereochilus marginatus* are not shown.

Analysis of the all-genes dataset produced a Bayesian consensus tree largely congruent with the *Cytb−Nd2−tRNA^trp^* phylogeny, again identifying the dwarf-Edwards clade and its two subclades (all PP = 1.0; Fig. 3). The two phylogenies differ only in their placement of 1) the *E. bislineata* and *E. longicauda* complexes (sister groups in the all-genes topology) and 2) the aforementioned *naufragia* + *tonkawae* pairing. Specifically, *naufragia* + *tonkawae* shift from being the sister group to the dwarf western lineage–weakly supported in the *Cytb−Nd2−tRNA^trp^* phylogeny (PP = 0.90; ML bootstrap  = 42%)–and become the sister group to the remaining Edwards Plateau species (PP = 1.0) in the all-genes phylogeny. ML analysis of the all-genes dataset produced a topology identical to the Bayesian consensus tree, with bootstrap values strongly supporting the dwarf-Edwards clade and its two subclades (Fig. 3).

### Species Trees

**Figure 4:**
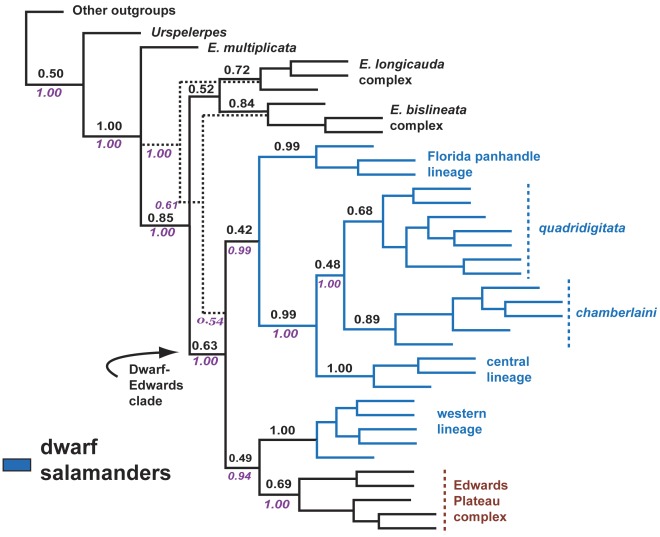
BCA and BEST species trees. Dotted lines depict alternative placement of the *Eurycea bislineata* complex identified in the BEST analysis. Numbers adjacent nodes are concordance factors (BCA) and BEST PP (italicized) values.

Figure 4 depicts the species trees generated by BCA for the five (mitochondrial gene partitioned) and three (mitochondrial genes combined) loci runs, which were topologically identical to the all-genes concatenation tree (Fig. 3). The BEST analysis produced a similar species tree, differing only in its placement of the *E. bislineata* complex, shown as the sister group to the dwarf-Edwards clade rather than the sister group to the *E. longicauda* complex (Fig. 4). Dwarf-Edwards relationships depicted in BCA and BEST trees were identical to those of the all-genes BI and ML trees (Fig. 3).

### Paraphyly through Introgression?

Could the sister group relationship observed between the dwarf western lineage and members of the Edwards Plateau complex represent historical introgression? If paraphyly were the result of hybridization, then digit evolution, whether loss or gain, could be called into question. Cases of historical introgression, as revealed by mitochondrial capture, include several amphibian examples [39]–[42]. However, the dwarf-Edwards clade does not bear a molecular phylogenetic signature indicative of introgression. The mitochondrial and all-genes trees are essentially congruent: each depicts dwarf salamanders as being paraphyletic, with the western lineage forming the sister group to the Edwards Plateau complex. Moreover, BI analyses of the nuclear genes alone (*Rag1, Pomc, Rag1*+*Pomc*; not illustrated) fail to recover a monophyletic dwarf salamander complex, as would be expected for mitochondrial capture via introgression [42], [43]. Both concordance (BCA) and coalescent (BEST) analyses yield the same dwarf-Edwards topology (i.e., the western lineage/Edwards Plateau complex is sister group to the remaining dwarf lineages) observed for the concatenated datasets. We would anticipate discordance among these estimated trees if hybridization (or, alternatively, coalescent variance) were a factor.

From a biological perspective, neither geographic distribution nor ecology presents opportunities for hybridization between the dwarf and Edwards complexes. They are presently allopatric: dwarf salamanders extend no further west than the San Jacinto drainage, and although the eastern-most species in the Edwards Plateau complex are found in the adjacent Brazos drainage, most occur south and west of the Colorado River (Fig. 1). The San Jacinto drainage roughly delimits the western range extent of eastern deciduous forest, which provides necessary habitat for dwarf salamanders. *Eurycea* is largely absent from the Brazos drainage, creating a distributional hiatus of ∼200 kilometers between the two complexes. The possibility of historic overlap notwithstanding, pronounced life history differences between the dwarf (terrestrial adults with terrestrially-based courtship) and Edwards Plateau (aquatic paedomorphs, many of which are subterranean and/or have extremely limited ranges) complexes would likely have preempted genetic exchange. For these reasons, we consider the observed paraphyly having arisen through introgression to be unlikely.

### Ancestral State Reconstruction

The MCMC ancestral state reconstruction provided marginal support (PP = 0.67) for a five-toed character state for the dwarf-Edwards node. Results from Bayes factor comparisons of the constraint hypotheses corroborate MCMC ancestral state reconstruction, offering marginal to strong statistical support for a five-toed character state (based on four independent runs, BF = 1.54–5.43; lnL *H*_parallelism_ = −10.106358/−8.37903; lnL *H*_re-evolution_ = −9.10535/−8.429534). The complementary results of these two analytical approaches favor, slightly, parallelism (two independent losses of the fifth toe among dwarf salamanders) over digit re-evolution as the more likely hypothesis for pedal evolution in the dwarf−Edwards clade.

## Discussion

Change in digit number within the dwarf-Edwards clade represents an otherwise rare evolutionary event in plethodontid salamanders, if not caudates in general. Nonetheless, certain chondrogenic features that distinguish caudate pedal development (relative to other tetrapods) provide plausible support for such change. First, salamanders undergo sequential digit formation during autopodial (hand/foot) development. Whereas frog and amniote digits develop synchronously, those of salamanders arise in a distinct anterior-to-posterior sequence: digit II then digit I develop first, followed by digits III, IV, and–on the pes–V [44]. The caudate mesopodium (carpal/tarsal elements) develops sequentially as well; elements proximal to digits I and II precede those proximal to digits III, IV, and V [45]. Second, the induction of digit loss in salamanders has shown that loss proceeds inversely from digit development [46], [47]. The fifth toe, the last digit to develop on the pes, is the first to disappear under experimental manipulation. This posterior-to-anterior polarity is mirrored in nature: the fifth toe is the digit most commonly lost, and evolutionary loss on the manus and pes proceeds from digits IV and V, respectively (Table 1). These observations indicate that the developmental pathways responsible for caudate digit formation are also conducive to evolutionary loss of the fifth toe.

Despite aforementioned differences, caudate digit development does respond to the patterning protein Sonic hedgehog (SHH) in a manner similar to that observed in other tetrapods. For example, manipulation of SHH expression readily induces sequential digit loss in the axolotl, *Ambystoma mexicanum* [47]. SHH provides as well a developmental explanation for evolutionary digit loss among closely related species in the scincid lizard genus *Hemiergis*: changes in digit number (2 fingers/2 toes, 3/3, 4/4, 5/5) correlate strongly with SHH temporal expression [48]. If temporal expression of SHH does specify differences within *Hemiergis*, then comparable SHH alterations could influence digit number variation in other closely related tetrapod species. Heterochronic changes in SHH expression (and attendant regulatory proteins such as GLI3 [49]) offer a tenable mechanism for parallel digit loss in dwarf salamanders or, alternatively, digit re-evolution in the Edwards Plateau complex.

### Conclusions

We provide phylogenetic evidence for an evolutionary change in digit number among members of the dwarf-Edwards clade in *Eurycea*, offering statistical support slightly favoring parallel loss of the fifth toe. We temper the later conclusion, however, by stressing that the results of our ancestral state reconstruction analyses do not constitute outright dismissal of digit re-evolution. Such a reversal would be the more remarkable outcome inasmuch as 1) digit re-evolution has not yet been documented in salamanders [15], and 2) the Edwards Plateau complex is exclusively paedomorphic–a developmental state viewed to be more influential in digit loss [9], [15] than gain. Increasingly, biologists identify the proximate mechanisms (i.e., genes involved, their precise mutations, specific effects on expression, etc.) that confer convergence [50], [51]. But unlike cases where natural selection drives such mechanisms [52], [53], the adaptive significance of toe loss in dwarf salamanders is not clear and instead may simply represent some form of developmental constraint. Indeed, it is altogether fitting that Wake’s [13] seminal paper on design limitation featured digit loss in salamanders as a putative case in point.

## Supporting Information

Table S1 Collection locality data for the dwarf salamander specimens.
(DOC)

Table S2 Primer sequences and amplification conditions.
(DOC)

Table S3 Genbank accession numbers, partitioned by dwarf phylogeographic lineages, other Eurycea, and outgroups. Museum/collector acronyms include: AUM  =  Auburn University Museum, DAB  =  David A. Beamer field series, JCM  =  John C. Maerz field series, NCSM  =  North Carolina State Museum, TNHC  =  Texas Natural History Collections, and USNM  =  U.S. National Museum. Numbers accompanying Edwards Plateau species correspond to collection localities in Fig. 1.
(DOC)
