# Supplementary material for: Digits Lost or Gained? Evidence for Pedal Evolution in the Dwarf Salamander Complex (Eurycea, Plethodontidae)
Source: PLoS One. 2012 May 23;7(5):e37544. doi: 10.1371/journal.pone.0037544 (PMC3359299; doi:10.1371/journal.pone.0037544)
Supplement: Table S1 — Collection locality data for the dwarf salamander specimens. (DOC) [file pone.0037544.s001.doc]

| **Lineage** | **ID #** | **State** | **County** |
| --- | --- | --- | --- |
| *chamberlaini* | 206 | NC | Craven |
| *chamberlaini* | 238 | NC | Halifax |
| *chamberlaini* | 288 | NC | Edgecombe |
| *chamberlaini* | 892 | NC | Craven |
| *chamberlaini* | 893 | SC | Colleton |
| *chamberlaini* | 976 | NC | Pitt |
| *chamberlaini* | 1532 | NC | Duplin |
| *chamberlaini* | 1776 | SC | Richland |
| *chamberlaini* | 1782 | SC | Calhoun |
| *chamberlaini* | 1867 | SC | Richland |
| *chamberlaini* | 2110 | SC | Colleton |
| *chamberlaini* | 2111 | SC | Colleton |
| *chamberlaini* | 2124 | SC | Anderson |
| *chamberlaini* | 2259 | SC | Richland |
| *chamberlaini* | 2777 | SC | Aiken |
| *chamberlaini* | 3824 | SC | Richland |
| *chamberlaini* | NCSM 75158 | NC | Wake |
| *chamberlaini* | NCSM 75389 | NC | Franklin |
| *quadridigitata* | 237 | NC | Robeson |
| *quadridigitata* | 269 | GA | Long |
| *quadridigitata* | 277 | SC | Berkeley |
| *quadridigitata* | 790 | FL | Liberty |
| *quadridigitata* | 850 | GA | Long |
| *quadridigitata* | 894 | SC | Colleton |
| *quadridigitata* | 995 | SC | Florence |
| *quadridigitata* | 1369 | GA | Coffee |
| *quadridigitata* | 1563 | FL | Putnam |
| *quadridigitata* | 1792 | SC | Jasper |
| *quadridigitata* | 1794 | SC | Hampton |
| *quadridigitata* | 1946 | SC | Jasper |
| *quadridigitata* | 2103 | SC | Florence |
| *quadridigitata* | 2271 | SC | Darlington |
| *quadridigitata* | 2442 | AL | Houston |
| *quadridigitata* | 2453 | FL | Duval |
| *quadridigitata* | 2471 | GA | Clinch |
| *quadridigitata* | 2473 | GA | Wayne |
| *quadridigitata* | 2510 | GA | Chatham |
| *quadridigitata* | 2546 | LA | St. Tammany |
| *quadridigitata* | 2568 | GA | Glynn |
| *quadridigitata* | 2752 | SC | Bamberg |
| *quadridigitata* | 2849 | SC | Orangeburg |
| *quadridigitata* | 3706 | SC | Jasper |
| *quadridigitata* | 3753 | SC | Clarendon |
| *quadridigitata* | 3769 | SC | Williamsburg |
| *quadridigitata* | 3825 | SC | Berkeley |
| *quadridigitata* | 3878 | SC | Florence |
| *quadridigitata* | 4653 | FL | Washington |
| *quadridigitata* | 4683 | FL | Jackson |
| *quadridigitata* | ACL 002 | GA | Coffee |
| central | 2326 | GA | Talbot |
| central | 2327 | AL | Conecuh |
| central | 2328 | AL | Bullock |
| central | 2333 | AL | Macon |
| central | 2345 | AL | Lowndes |
| central | 2351 | AL | Clarke |
| central | 2447 | GA | Marion |
| central | 4652 | FL | Washington |
| central | AU 37783 | AL | Conecuh |
| central | 9996 | GA | Emanuel |
| central | JCM 011 | GA | Talbot |
| FL panhandle | 246 | MS | Stone |
| FL panhandle | 2343 | AL | Covington |
| FL panhandle | 2348 | AL | Baldwin |
| FL panhandle | 2353 | AL | Baldwin |
| FL panhandle | 2444 | AL | Baldwin |
| western | 262 | TX | San Jacinto |
| western | 651 | LA | Grant |
| western | 652 | LA | Grant |
| western | 725 | TX | Nacogdoches |
| western | 747 | TX | Polk |
| western | 765 | TX | Tyler |
| western | 770 | TX | Newton |
| western | 775 | TX | Newton |
| western | 776 | TX | Newton |
| western | 777 | TX | Newton |
| western | 778 | TX | Newton |
| western | 779 | TX | Newton |
| western | 2139 | AR | Columbia |
| western | 2182 | LA | Tangipahoa |
| western | 2218 | LA | West Feliciana |
| western | 2435 | LA | Catahoula |
| western | 2539 | LA | St. Tammany |
| western | 4190 | LA | Natchitoches |
| western | 4208 | LA | Natchitoches |
| western | 4211 | LA | De Soto |
| western | 4225 | AR | Ouachita |
| western | 4231 | LA | Rapides |
| western | 4238 | AR | Columbia |

Unless otherwise noted, specimen ID numbers are DAB field series

numbers. Contact D. A. Beamer for more detailed locality data.
