# Supplementary material for: Digits Lost or Gained? Evidence for Pedal Evolution in the Dwarf Salamander Complex (Eurycea, Plethodontidae)
Source: PLoS One. 2012 May 23;7(5):e37544. doi: 10.1371/journal.pone.0037544 (PMC3359299; doi:10.1371/journal.pone.0037544)
Supplement: Table S2 — Primer sequences and amplification conditions. (DOC) [file pone.0037544.s002.doc]

| Gene | Primer name | Sequence (5’-3’) | Temp,cycle# | Reference |
| --- | --- | --- | --- | --- |
| *Cytb* | MVZ15 | GAACTAATGGCCCACACWWTACGNAA | 50^0^C, 35 | 54 |
|  | quad *b* r | TGGTCCAATCTCAATAAATGGGGGTTC |  | This study |
|  | quad *b* int. | TCAGTAGAYAAAGCAACACT |  | This study |
| *Nd2* | L4437 | AAGCTTTCGGGCCCATACC | 50^0^C, 35 | 55 |
|  | Eu*Co* r | CTTTRTGGTTTGTTGARAATAGTCATCG |  | This study |
|  | quad *nd* int. | TGACARAARYTAGCCCCAATA |  | This study |
| *16s* | 16S-286 | AGATAGAAACCGACCTGGAT | 50^0^C, 35 | 56 |
|  | 16S-381 | ACCCCGCCTGTTTACCAAAAACAT |  | 56 |
| *Rag1* | Eu Rag f | AACTGGACGRCAGATTTTCCAGCCCTTACA | 58^0^C, 45 | This study |
|  | Eu Rag r2 | GCCCATTAGYTCACGCAGAGCCTCTCG |  | This study |
|  | quad Rag int. | GTACMCTGTGTGATTCTA |  | This study |
| *Pomc* | POMCAmphF | ATATGTCATGAGCCATTTTCGCTGGAA | 58^0^C, 45 | 57 |
|  | POMCAmphR | GGCATTTTTGAAAAGAGTCATTAGAGG |  | 57 |
