# Supplementary material for: Digits Lost or Gained? Evidence for Pedal Evolution in the Dwarf Salamander Complex (Eurycea, Plethodontidae)
Source: PLoS One. 2012 May 23;7(5):e37544. doi: 10.1371/journal.pone.0037544 (PMC3359299; doi:10.1371/journal.pone.0037544)
Supplement: Table S3 — Genbank accession numbers, partitioned by dwarf phylogeographic lineages, other Eurycea, and outgroups. Museum/collector acronyms include: AUM = Auburn University Museum, DAB = David A. Beamer field series, JCM = John C. Maerz field series, NCSM = North Carolina State Museum, TNHC = Texas Natural History Collections, and USNM = U.S. National Museum. Numbers accompanying Edwards Plateau species correspond to collection localities in Fig. 1. (DOC) [file pone.0037544.s003.doc]

| **Species or lineage** | **ID #** | ***Cytb*** | ***Nd2*** | ***16s*** | ***Rag1*** | ***Pomc*** |
| --- | --- | --- | --- | --- | --- | --- |
| *chamberlaini* | DAB 893 | JQ920707 | JQ920891 | JQ920611 | JQ920795 | JQ920756 |
| *chamberlaini* | DAB 1776 | JQ920700 | JQ920884 | JQ920608 | JQ920792 | JQ920753 |
| *chamberlaini* | DAB 1867 | JQ920702 | JQ920886 | JQ920609 | JQ920793 | JQ920754 |
| *chamberlaini* | DAB 2124 | JQ920705 | JQ920889 | JQ920610 | JQ920794 | JQ920755 |
| *chamberlaini* | NCSM 75158 | JQ920697 | JQ920881 | JQ920607 | JQ920791 | JQ920752 |
| *chamberlaini* | DAB 206 | JQ920695 | JQ920879 |  |  |  |
| *chamberlaini* | DAB 976 | JQ920694 | JQ920878 |  |  |  |
| *chamberlaini* | DAB 1532 | JQ920699 | JQ920883 |  |  |  |
| *chamberlaini* | DAB 1782 | JQ920701 | JQ920885 |  |  |  |
| *chamberlaini* | DAB 2110 | JQ920708 | JQ920892 |  |  |  |
| *chamberlaini* | DAB 2111 | JQ920709 | JQ920893 |  |  |  |
| *chamberlaini* | DAB 2259 | JQ920703 | JQ920887 |  |  |  |
| *chamberlaini* | DAB 2777 | JQ920706 | JQ920890 |  |  |  |
| *chamberlaini* | DAB 3824 | JQ920704 | JQ920888 |  |  |  |
| *chamberlaini* | NCSM 75389 | JQ920696 | JQ920880 |  |  |  |
| *quadridigitata* | ACL 002 | JQ920665 | JQ920849 | JQ920601 | JQ920785 | JQ920746 |
| *quadridigitata* | DAB 277 | JQ920680 | JQ920864 | JQ920606 | JQ920790 | JQ920751 |
| *quadridigitata* | DAB 2442 | JQ920669 | JQ920853 | JQ920604 | JQ920788 | JQ920749 |
| *quadridigitata* | DAB 2453 | JQ920668 | JQ920852 | JQ920602 | JQ920786 | JQ920747 |
| *quadridigitata* | DAB 2546 | JQ920672 | JQ920856 | JQ920600 | JQ920784 | JQ920745 |
| *quadridigitata* | DAB 3878 | JQ920679 | JQ920863 | JQ920605 | JQ920789 | JQ920750 |
| *quadridigitata* | DAB 4653 | JQ920671 | JQ920855 | JQ920603 | JQ920787 | JQ920748 |
| *quadridigitata* | DAB 237 | JQ920676 | JQ920860 |  |  |  |
| *quadridigitata* | DAB 269 | JQ920661 | JQ920845 |  |  |  |
| *quadridigitata* | DAB 790 | JQ920673 | JQ920857 |  |  |  |
| *quadridigitata* | DAB 850 | JQ920662 | JQ920846 |  |  |  |
| *quadridigitata* | DAB 894 | JQ920688 | JQ920872 |  |  |  |
| *quadridigitata* | DAB 995 | JQ920681 | JQ920865 |  |  |  |
| *quadridigitata* | DAB 1369 | JQ920664 | JQ920848 |  |  |  |
| *quadridigitata* | DAB 1563 | JQ920675 | JQ920859 |  |  |  |
| *quadridigitata* | DAB 1792 | JQ920690 | JQ920874 |  |  |  |
| *quadridigitata* | DAB 1794 | JQ920686 | JQ920870 |  |  |  |
| *quadridigitata* | DAB 1946 | JQ920687 | JQ920871 |  |  |  |
| *quadridigitata* | DAB 2103 | JQ920677 | JQ920861 |  |  |  |
| *quadridigitata* | DAB 2271 | JQ920682 | JQ920866 |  |  |  |
| *quadridigitata* | DAB 2471 | JQ920667 | JQ920851 |  |  |  |
| *quadridigitata* | DAB 2473 | JQ920666 | JQ920850 |  |  |  |
| *quadridigitata* | DAB 2510 | JQ920689 | JQ920873 |  |  |  |
| *quadridigitata* | DAB 2568 | JQ920663 | JQ920847 |  |  |  |
| *quadridigitata* | DAB 2752 | JQ920685 | JQ920869 |  |  |  |
| *quadridigitata* | DAB 2849 | JQ920683 | JQ920867 |  |  |  |
| *quadridigitata* | DAB 3706 | JQ920692 | JQ920876 |  |  |  |
| *quadridigitata* | DAB 3753 | JQ920678 | JQ920862 |  |  |  |
| *quadridigitata* | DAB 3769 | JQ920693 | JQ920877 |  |  |  |
| *quadridigitata* | DAB 3825 | JQ920684 | JQ920868 |  |  |  |
| *quadridigitata* | DAB 4332 | JQ920691 | JQ920875 |  |  |  |
| *quadridigitata* | DAB 4683 | JQ920674 | JQ920858 |  |  |  |
| central lineage | AU 37783 | JQ920710 | JQ920894 | JQ920612 | JQ920796 | JQ920757 |
| central lineage | DAB 2327 | JQ920711 | JQ920895 | JQ920613 | JQ920797 | JQ920758 |
| central lineage | JCM 011 | JQ920720 | JQ920904 | JQ920614 | JQ920798 | JQ920759 |
| central lineage | DAB 2326 | JQ920718 | JQ920902 |  |  |  |
| central lineage | DAB2328 | JQ920712 | JQ920896 |  |  |  |
| central lineage | DAB 2333 | JQ920716 | JQ920900 |  |  |  |
| central lineage | DAB 2345 | JQ920713 | JQ920897 |  |  |  |
| central lineage | DAB 2351 | JQ920715 | JQ920899 |  |  |  |
| central lineage | DAB 2447 | JQ920719 | JQ920903 |  |  |  |
| central lineage | DAB 4652 | JQ920714 | JQ920898 |  |  |  |
| central lineage | DAB 9996 | JQ920717 | JQ920901 |  |  |  |
| western lineage | DAB 765 | JQ920639 | JQ920823 | JQ920595 | JQ920779 | JQ920740 |
| western lineage | DAB 2539 | JQ920654 | JQ920838 | JQ920598 | JQ920782 | JQ920743 |
| western lineage | DAB 4211 | JQ920660 | JQ920844 | JQ920597 | JQ920781 | JQ920742 |
| western lineage | DAB 4225 | JQ920648 | JQ920832 | JQ920596 | JQ920780 | JQ920741 |
| western lineage | DAB 4231 | JQ920656 | JQ920840 | JQ920599 | JQ920783 | JQ920744 |
| western lineage | DAB 262 | JQ920649 | JQ920833 |  |  |  |
| western lineage | DAB 652 | JQ920650 | JQ920834 |  |  |  |
| western lineage | DAB 725 | JQ920643 | JQ920827 |  |  |  |
| western lineage | DAB 747 | JQ920651 | JQ920835 |  |  |  |
| western lineage | DAB 770 | JQ920644 | JQ920828 |  |  |  |
| western lineage | DAB 776 | JQ920640 | JQ920824 |  |  |  |
| western lineage | DAB 777 | JQ920641 | JQ920825 |  |  |  |
| western lineage | DAB 778 | JQ920642 | JQ920826 |  |  |  |
| western lineage | DAB 779 | JQ920658 | JQ920842 |  |  |  |
| western lineage | DAB 2139 | JQ920645 | JQ920829 |  |  |  |
| western lineage | DAB 2182 | JQ920652 | JQ920836 |  |  |  |
| western lineage | DAB 2218 | JQ920653 | JQ920837 |  |  |  |
| western lineage | DAB 2435 | JQ920655 | JQ920839 |  |  |  |
| western lineage | DAB 4190 | JQ920659 | JQ920843 |  |  |  |
| western lineage | DAB 4208 | JQ920646 | JQ920830 |  |  |  |
| western lineage | DAB 4238 | JQ920647 | JQ920831 |  |  |  |
| FL panhandle lineage | DAB 246 | JQ920634 | JQ920818 | JQ920592 | JQ920776 | JQ920737 |
| FL panhandle lineage | DAB 2343 | JQ920635 | JQ920819 | JQ920593 | JQ920777 | JQ920738 |
| FL panhandle lineage | DAB 2353 | JQ920637 | JQ920821 | JQ920594 | JQ920778 | JQ920739 |
| FL panhandle lineage | DAB 2348 | JQ920636 | JQ920820 |  |  |  |
| FL panhandle lineage | DAB 2444 | JQ920638 | JQ920822 |  |  |  |
| *E. bislineata* | DAB 3260 | JQ920620 | JQ920804 | JQ920581 | JQ920765 | JQ920726 |
| *E. cirrigera* | DAB 2214 | JQ920622 | JQ920806 | JQ920583 | JQ920767 | JQ920728 |
| *E. guttolineata* | DAB 990 | JQ920625 | JQ920809 | JQ920586 | JQ920770 | JQ920731 |
| *E. latitans* (#6) | TNHC 64504 | JQ920628 | JQ920812 |  |  |  |
| *E.longicauda* | DAB 9995 | JQ920624 | JQ920808 | JQ920585 | JQ920769 | JQ920730 |
| *E.lucifuga* | DAB 2152 | JQ920623 | JQ920807 | JQ920584 | JQ920768 | JQ920729 |
| *E. multiplicata* | DAB 4127 | JQ920619 | JQ920803 | JQ920580 | JQ920764 | JQ920725 |
| *E. nana* (#3) | TNHC 52758 | JQ920630 | JQ920814 | JQ920590 | JQ920774 | JQ920735 |
| *E. naufragia* (#1) | TNHC 51013 | JQ920627 | JQ920811 | JQ920588 | JQ920772 | JQ920733 |
| *E. neotenes* (#7) | DMH 90-91 | JQ920633 | JQ920817 |  |  |  |
| *E. petrophila* (#5) | TNHC 51120 | JQ920632 | JQ920816 |  |  |  |
| *E. sosorum* (#4) | TNHC 51183 | JQ920631 | JQ920815 | JQ920591 | JQ920775 | JQ920736 |
| *E. tonkawae* (#2) | DAB 4656 | JQ920626 | JQ920810 | JQ920587 | JQ920771 | JQ920732 |
| *E. troglodytes* (#8) | TNHC 51086 | JQ920629 | JQ920813 | JQ920589 | JQ920773 | JQ920734 |
| *E. wilderae* | DAB 1893 | JQ920621 | JQ920805 | JQ920582 | JQ920766 | JQ920727 |
| *U. brucei* | USNM55823 | JQ920618 | JQ920802 | JQ920579 | JQ920763 | JQ920724 |
| *G. porphyriticus* | DAB 9999 | JQ920616 | JQ920800 | JQ920577 | JQ920761 | JQ920722 |
| *P. ruber* | DAB 9997 | JQ920615 | JQ920799 | JQ920576 | JQ920760 | JQ920721 |
| *S. marginatus* | DAB 9998 | JQ920617 | JQ920801 | JQ920578 | JQ920762 | JQ920723 |
